# Perfusion Bioreactor Conditioning of Small-diameter Plant-based Vascular Grafts

**DOI:** 10.1007/s13770-024-00670-0

**Published:** 2024-10-01

**Authors:** Nicole Gorbenko, John C. Vaccaro, Ryan Fagan, Robert A. Cerro, Jonah M. Khorrami, Lucia Galindo, Nick Merna

**Affiliations:** 1 Bioengineering Program, Fred DeMatteis School of Engineering and Applied Science, Hofstra University, 229 Science and Innovation Center, Hempstead, NY 11549 USA; 2 Mechanical Engineering Program, Fred DeMatteis School of Engineering and Applied Science, Hofstra University, 200B Weed Hall, Hempstead, NY 11549 USA; 3 Fred DeMatteis School of Engineering and Applied Science, Hofstra University, 016 Adams Hall, Hempstead, NY 11549 USA

**Keywords:** Bioreactor, Vascular graft, Plant, Tissue engineering

## Abstract

**Background:**

Vascular grafts are mainly composed of synthetic materials, but are prone to thrombosis and intimal hyperplasia at small diameters. Decellularized plant scaffolds have emerged that provide promising alternatives for tissue engineering. We previously developed robust, endothelialized small-diameter vessels from decellularized leatherleaf viburnum. This is the first study to precondition and analyze plant-based vessels under physiological fluid flow and pressure waveforms. Using decellularized leatherleaf viburnum as tissue-engineered grafts for implantation can have profound impacts on healthcare due to their biocompatibility and cost-effective production.

**Methods:**

A novel perfusion bioreactor was designed, capable of accurately controlling fluid flow rate and pressure waveforms for preconditioning of small-diameter vascular grafts. A closed-loop system controlled pressure waveforms, mimicking physiological values of 50–120 mmHg at a frequency of 8.75 Hz for fluid flow reaching 5 mL/min. Plant-based vascular grafts were recellularized with endothelial and vascular smooth muscle cells and cultured for up to 3 weeks in this bioreactor. Cell density, scaffold structure and mechanics, thrombogenicity, and immunogenicity of grafts were evaluated.

**Results:**

Bioreactor treatment with fluid flow significantly increased luminal endothelial cell density, while pressure waveforms reduced thrombus formation and maintained viable vascular smooth muscle cells within inner layers of grafts compared to static controls. Suture retention of grafts met transplantation standards and white cell viability was suitable for vascular remodeling.

**Conclusion:**

Low thrombogenicity of endothelialized leatherleaf viburnum holds great potential for vascular repair. This study provides insight into benefits of conditioning plant-based materials with hemodynamic forces at higher frequencies that have not previously been investigated.

**Supplementary Information:**

The online version contains supplementary material available at 10.1007/s13770-024-00670-0.

## Introduction

Cardiovascular disease (CVD) is the leading cause of death globally, and results in a loss of $203 billion in annual income in the United States [1, 2]. CVD can block coronary arteries, which often requires coronary bypass to redirect blood flow. However, limited availability of autologous vessels for this procedure creates a need for small-diameter (< 6 mm) vascular grafts derived from synthetic or natural materials.

Vascular grafts are mainly composed of GORE-TEX (expanded polytetrafluoroethylene) or Dacron (polyethylene terephthalate), but after six decades since their introduction, they have had limited success due to thrombosis, poor patency, and intimal hyperplasia at small diameters. Thrombosis often occurs due to the absence of endothelial cells in the graft lumen and activation of clotting mechanisms [3]. Intimal hyperplasia is caused by migration of vascular smooth muscle cells from the graft media to lumen, suture line stress concentrations, and compliance mismatch [4, 5]. Thus, a successful tissue-engineered vascular graft must possess adequate mechanical strength, an endothelialized lumen to minimize thrombus formation, and low cytotoxicity to minimize the immunogenic response.

Researchers have identified many promising natural materials for vascular applications, such as collagen, silk, chitosan and cellulose [4]. With recent advancements in plant decellularization, plant-derived cellulose scaffolds have emerged that mimic the structure and mechanics of collagen and elastin fibers found in native tissue. This has made decellularized plants good candidates for skeletal, cardiac, bone, and vascular tissue engineering [2, 6]. These plant-based scaffolds have previously been seeded with various types of mammalian cells and have been shown to be cost-effective, biocompatible, and mechanically suitable. Decellularization utilizes harsh chemical reagents to remove foreign DNA that may cause an immune response. Removal of the cellular material leaves an extracellular matrix (ECM) which has been shown to help guide cell behavior upon recellularization. The decellularization process aims to preserve the tissue’s structural and mechanical properties in order to replicate the microenvironment needed to support engineered vascular tissues. The effects of collagen fiber structure on cell behavior and tissue mechanics have been previously investigated, such as the relationship between fiber density, alignment and mechanical properties [7, 8]. Similarly, cellulose fiber structure of decellularized plant tissue could also affect important graft properties but it remains relatively unexplored. Adequate suture retention force is essential for securing vascular grafts during implantation, preventing leakage and withstanding the long-term forces within the circulatory system. Suture retention testing can help determine if the graft can withstand the tensile load of sutures during implantation, without rupturing or scattering [9]. Pore size of the decellularized scaffold is another important property, with an optimal diameter of 5–500 µm [10]. Pores that are too small may prevent cellular infiltration but can cause blood leakage if too large. As potential immunogenicity of plant-derived scaffolds remains another key challenge, white cell viability for decellularized scaffolds should be evaluated to quantify the immune cell cytotoxicity *in vitro* [11]. A reduction in white cell viability would suggest that a biomaterial is not able to optimally support wound healing *in vivo*. While decellularized plants are promising tools to successfully tissue engineer vascular grafts, it is essential to verify that they meet all of these requirements: suitable mechanical properties, appropriate pore size, and minimal immune response.

It is well established that preconditioning of tissue-engineered vascular grafts with physiological fluid flow and pressure improves endothelial cell and vascular smooth muscle cell performance and mechanical properties of the tissue [12]. However, the use of bioreactors to condition plant-derived scaffolds has been limited to laboratory grown meat. Within 24 h, application of laminar shear stress stimulates endothelial cells to align with flow and form a functional endothelium, which helps prevent loss of the endothelial cells and thrombosis in implanted grafts [13]. Long-term culture of 2–4 weeks helps form a continuous and mature endothelial layer that can resist shear stress and prevent thrombosis, enhancing graft patency after implantation. When exposed to physiological shear stress found in healthy human arteries, endothelial cells express anti-inflammatory molecules that prevent atherosclerosis [14]. On the other hand, cyclic circumferential strain mimics the pulsatile nature of blood flow and regulates vascular smooth muscle cell phenotype and ECM production, generating more robust tissue-engineered vascular grafts with increased suture retention and burst pressure. Additionally, pulsatile flow increases alignment and contractility of vascular smooth muscle cell [15]. Pre-conditioning under pulsatile flow for an extended period, such as 3 weeks, has been demonstrated to improve cell viability and prepare vascular smooth muscle cells to respond to cyclic strain *in vivo* which is critical for long-term functionality of the graft. Many bioreactors have been developed to deliver the mechanical stimuli described above for vascular tissue engineering, but none have tried to incorporate higher frequency pressure waveforms (above 2 Hz). Most systems use peristaltic rollers, which are known for generating accurate flow rate control [16]. However, peristaltic rollers are inherently pulsatile, where flow rate is proportional to pulse rate, and it is very difficult to control the rates independently. Wolf et al. generated tunable pulse frequency ranging from 0.5 to 2 Hz, but pressure and flow lacked consistency at higher frequencies [17]. Similarly, others have decoupled effects of shear stress and cyclic pressure, inducing circumferential stretch at a fixed frequency of 0.5 Hz [18, 19]. Together, fluid flow and pressure-induced stretch increase spatial complexity of co-culture bioreactors and better mimic native vasculature which enhances growth and maturation of tissue-engineered vascular grafts [20, 21]. Therefore, a bioreactor capable of decoupling flow and pressure at higher frequencies is important to identify preconditioning methods that can enhance the performance of vascular grafts.

We previously developed robust, endothelialized vessels 2 mm in diameter from sodium dodecyl sulfate (SDS)-decellularized leatherleaf viburnum (*Viburnum rhytidophyllum)*, gelatin, and glutaraldehyde [22]. By decreasing the treatment time and concentration of clearing solution, our method of decellularization fully removed foreign DNA to minimize foreign body response, while better preserving the mechanical integrity of the leaf scaffold when compared to other decellularization protocols [2, 23]. These grafts had a modulus comparable to native blood vessels, a higher tensile stress and failure strain, and maintained physiological burst pressure for 90 days *in vitro*.

In this study, we designed a bioreactor system to precondition these plant-based grafts for up to 3 weeks with appropriate physiological stimuli to provide insight into effects of hemodynamic forces. Treated grafts demonstrated reduced thrombosis, increased endothelial cell density and preservation of material strength and structure *in vitro*. This marks the first time that a bioreactor has been used to precondition decellularized plant scaffolds for tissue repair. Here we sought to characterize the structural properties, thrombogenicity, and recellularization potential of decellularized leatherleaf viburnum following bioreactor treatment to determine the suitability of plant-based materials for vascular repair.

## Materials and methods

To analyze the potential of plant-based grafts for vascular repair, the grafts were preconditioned at pressures of 50–120 mmHg and a flow rate of 5 mL/min to test the effect of endothelialization on thrombogenicity. The grafts’ structural properties were evaluated using a scanning electron microscope (SEM) and histology, their mechanical strength was measured using suture retention testing, and immunogenicity was assessed by white cell viability.

### Pressure controlled bioreactor

**Fig. 1:**
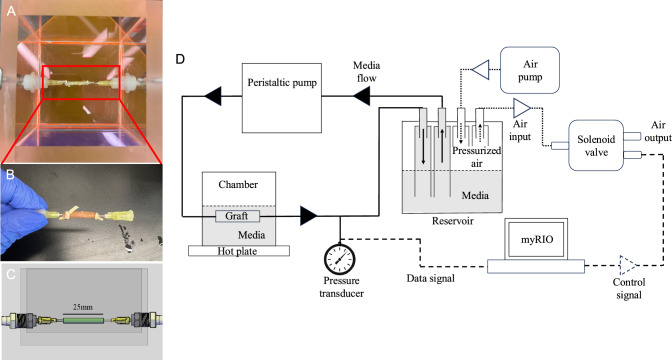
**A** Representative photo of culture region in chamber. **B** Representative photo of the vascular graft. **C** Side view of graft in chamber in AutoCAD. **D** Schematic of bioreactor system with its individual components

The bioreactor system developed for mechanical preconditioning of vascular grafts is shown in Fig. 1. A Masterflex L/S standard digital drive variable speed peristaltic pump fitted with a L/S Easy Load-II pump head (Cole-Parmer) generated a flow rate of up to 5 mL/min. Fluid passed through a L/S 15 High-Performance Precision Pump C-Flex tubing into a 100 mL reservoir sealed with a rubber stopper with four 4.76 mm barbs passing through (Fig. 1). Inside the reservoir, four pieces of tubing were attached to barbs. Two tubes were submerged in media to enable fluid flow, while the other two tubes were above the media level to regulate the reservoir pressure as will be described in Sect. 2.6. Air exchange in the reservoir occurred through a 0.22 μm filter. The reservoir connected to a 11.4 × 11.4 × 11.4 cm clear acrylic culture chamber (Fig. 1A, C). The walls had threaded holes for straight, polypropylene 6.35 mm NPT male single barb tubing fitting connectors outside the chamber and polypropylene plastic Luer-lock 3.175 mm hose ID connectors inside for graft cannulation.

### Feedback system setup

The closed-loop control feedback system was designed to expose grafts in the bioreactor to physiological pressure waveforms while maintaining long-term sterility. This was achieved by pressurizing the air trapped within the reservoir. The system consisted of a GREDIA 1/2" DC 12 V solenoid valve (normally closed water inlet flow switch), G1/4" pressure transducer (0–517 mmHg 1% full scale accuracy), Busch R5 RA 0063 Rotary Vane Air Pump (124 mmHg, 15 L/min flowrate), and National Instruments myRIO (Fig. 1D). The pressure transducer was connected to a tee at the chamber outlet. The solenoid valve and air pump were connected to two reservoir barbs not submerged in media. The wiring diagram of the pressure transducer and solenoid valve are depicted in Online Resource 1A.

A custom LabVIEW Virtual Instrument continuously monitored the bioreactor outlet pressure and controlled the solenoid valve opening to maintain 50–120 mmHg at a frequency of 8.75 Hz to replicate rat physiology (Online Resource 1). [24]. The measured pressure waveform frequency was adjusted by changing the air pump flow rate and the timing of the opening and closing of the solenoid valve.

### Cell culture

Cryopreserved primary rat aortic endothelial cells and vascular smooth muscle cells from Cell Applications (San Diego, CA, USA) were thawed at passage three and passage two and expanded in Heracell 150i CO_2_ incubators (Thermo Fisher Scientific, Waltham, MA, USA) with 5% CO_2_, and used up to passage eight and sixteen, respectively. Endothelial cells were cultured in Rat Endothelial Cell Growth Medium (Cell Applications) supplemented with 2% fetal calf serum, endothelial growth supplement, epidermal growth factor, basic fibroblast growth factor, heparin, and hydrocortisone. Vascular smooth muscle cells were cultured in Smooth Muscle Cell Growth Medium (Cell Applications) and differentiated in Smooth Muscle Cell Induction Medium (Cell Applications).

### Fabrication of grafts

Fresh leatherleaf viburnum was decellularized for 72 h in 2% SDS (Sigma-Aldrich, St. Louis, MO, USA) followed by 6 h in a clearing solution of 10% bleach and 0.1% Triton X-100 (Sigma-Aldrich) and sterilized for 1 h in 70% ethanol as previously described [22]. SDS-decellularized leatherleaf cut to 21 × 25 mm was coated with fibronectin (10 μg/mL, Sigma-Aldrich), seeded with 15,000 vascular smooth muscle cells/cm^2^ on the abaxial surface and incubated at 37 °C for 24 h. The recellularized scaffolds were wrapped around a stainless-steel rod 1 mm in diameter, using the adaxial side of leaves as the inner surface of the graft (Online Resource 2). A measure of 44.1 μL of 50% bovine gelatin (Sigma-Aldrich) heated to 55 °C, mixed with 5.9 μL of 25% glutaraldehyde (Sigma-Aldrich), was applied evenly across the leaf’s width at one end to maximize viability of the vascular smooth muscle cells. The leaf was wrapped around the rod, held for 1 min to allow the gelatin to cross-link, and incubated in media at 37 °C for 4 h. Recellularized 3D grafts were filled with fibronectin with 20-gauge needle syringe barb adapters attached at both ends and incubated for 1 h. A measure of 946,000 endothelial cells/cm^2^ was injected into the lumen of each graft. Seeded grafts were incubated at 37 °C in cell culture media and rotated 90° every 15 min for 3 h. After 24 h, select recellularized grafts were fixed in 4% formaldehyde (Sigma-Aldrich) and stained with Hoechst 33,342 (Thermo Fisher Scientific) for 30 s. Blinded counters manually recorded nuclei in three confluent regions on each sample (N = 9) using fluorescent microcopy (FV3000 scanning confocal microscope, Olympus Corporation, Shinjuku City, Tokyo, Japan) at 20 × magnification. Additional samples were trypsinized and adhered cells were counted.

### Bioreactor setup

Prior to use, the chamber, reservoir, and tubing were sterilized with 10% bleach for 10 min and rinsed with deionized water. Components were then sterilized with 70% ethanol and assembled in a biosafety cabinet. The reservoir was filled with Medium 199 (M199, Sigma-Aldrich), 2% fetal bovine serum (FBS, BioTC, Wayne, NJ, USA), and 1X penicillin–streptomycin (Thermo Fisher Scientific), connected to the rubber stopper and tubing, primed with media, and connected to the chamber as shown in Fig. 1. Grafts were cannulated with 20-gauge needles and tied with rubber bands. A recellularized 3D graft was placed into the chamber by twisting onto Luer-lock barbs and submerged in 250 mL M199 with 2% FBS.

### Flow and pressure regimen

Seeded grafts were mechanically preconditioned with one of two pathways: (1) 5 mL/min fluid flow or (2) fluid flow plus a 50–120 mmHg pressure waveform (N = 3). Fluid flow was gradually increased from 0.2 mL/min to 5 mL/min over 4 h to better simulate the microenvironments of fetal to adult rats, allowing the cells to adapt to the increasing forces. The feedback system gradually introduced an 8.75 Hz pressure waveform. After 24 h or after 3 weeks, grafts preconditioned with fluid flow were fixed with 4% formaldehyde and stained with Hoechst or trypsinized for cell counting to assess changes in luminal endothelial cell density in response to fluid flow.

For laminar flow of Newtonian fluids, wall shear stress in a tube can be calculated using the Poiseuille equation: $\tau =\frac{4Q\mu }{\pi {r}^{3}}$ where Q is volumetric flow rate, µ is dynamic viscosity of the media and r is inner radius of the graft. For media viscosity µ of 6.92 × 10^–4^ N m^2^/s, a flow rate of 5 mL/min generated a mean wall shear stress of 0.587 Pa within the nominal 1 mm inner diameter vascular grafts. Considering the pulsatile nature of the flow and change in tube diameter, the temporally and spatially averaged fluid velocity was estimated to be 10.6 cm/s, resulting in a Reynolds number (scaled by the nominal inner diameter of 1 mm) of 152. Entry length for laminar flow was estimated to be 9 mm. As the graft was 13.5 mm away from a diameter change, the entire graft was exposed to fully developed laminar flow. The bioreactor generated a pressure of 50–120 mmHg at up to 8.75 Hz (mean pressure of 89 mmHg) to simulate animal or human models (Online Resource 1). We adjusted pressure and frequency to gradually introduce mechanical stimulation.

### Cell viability

A live/dead stain kit (#R37601, Invitrogen, Waltham, MA, USA) was used to evaluate viability of vascular smooth muscle cells in grafts after 24 h and 3 weeks of treatment with flow in the bioreactor. Viable cells were counted (N = 9) and the ratio of viable cells to total cells was calculated as previously described [25].

### *In vitro* thrombosis assay

To assess the effects of endothelialization and bioreactor conditioning on the thrombogenicity of the plant-based grafts, acellular and recellularized grafts under static conditions or with 24 h of bioreactor feedback preconditioning with flow and pressure were cannulated and perfused with whole rat blood using a syringe pump (N = 3). Fresh whole rat blood in 4.5 mL citrate tubes was combined with Alexa Fluor 546-labeled fibrinogen from human plasma (Thermo Fisher Scientific) at a concentration of 28 μg/mL, as previously described [26]. Blood was recalcified with 0.2 M calcium chloride then injected into grafts for 30 min at 78.3 μL/min. Grafts were rinsed with phosphate-buffered saline (PBS), fixed with 4% formaldehyde, and stained with Hoechst. Thrombus-free area fraction and thrombus thickness were assessed by measuring and subtracting background from 16-bit images and auto thresholding platelet and fibrinogen images using ImageJ’s Maximum Entropy plugin.

### Histological staining

Grafts before and after 24 h of bioreactor preconditioning with physiological pressure and flow were fixed in 4% formaldehyde for 1 h and sent to VitroVivo Biotech (Rockville, MD, USA) for paraffin embedding and hematoxylin and eosin (H&E) staining. Brightfield microscopy was performed at 40 × magnification, and a tile scan was assembled (Olympus FV3000). The distribution of ECM orientation, wall thickness, fiber thickness and cell wall fiber volume-fraction were measured in ImageJ for 3 images per sample (N = 9).

### Scanning electron microscopy

Sections of acellular and recellularized grafts before and after 24 h of bioreactor preconditioning with flow and pressure treatments were fixed with 2.5% glutaraldehyde and rinsed with ascending ethanol concentrations (70–100%) for 1 h. Samples were dried in a Samdri-795 critical point dryer (Tousimis, Rockville, MD, USA), mounted on aluminum stubs, and coated with gold using an EMS-550 sputter coater. A Quanta FEI-250 SEM imaged the samples at 110 × and 800 × magnification. Inner diameter, outer diameter and pore size were calculated to assess patency and structure of the grafts. For each sample, three SEM images were analyzed (N = 9). For each image, at least 10 pores were manually measured using ImageJ.

### Suture retention testing

**Fig. 2:**
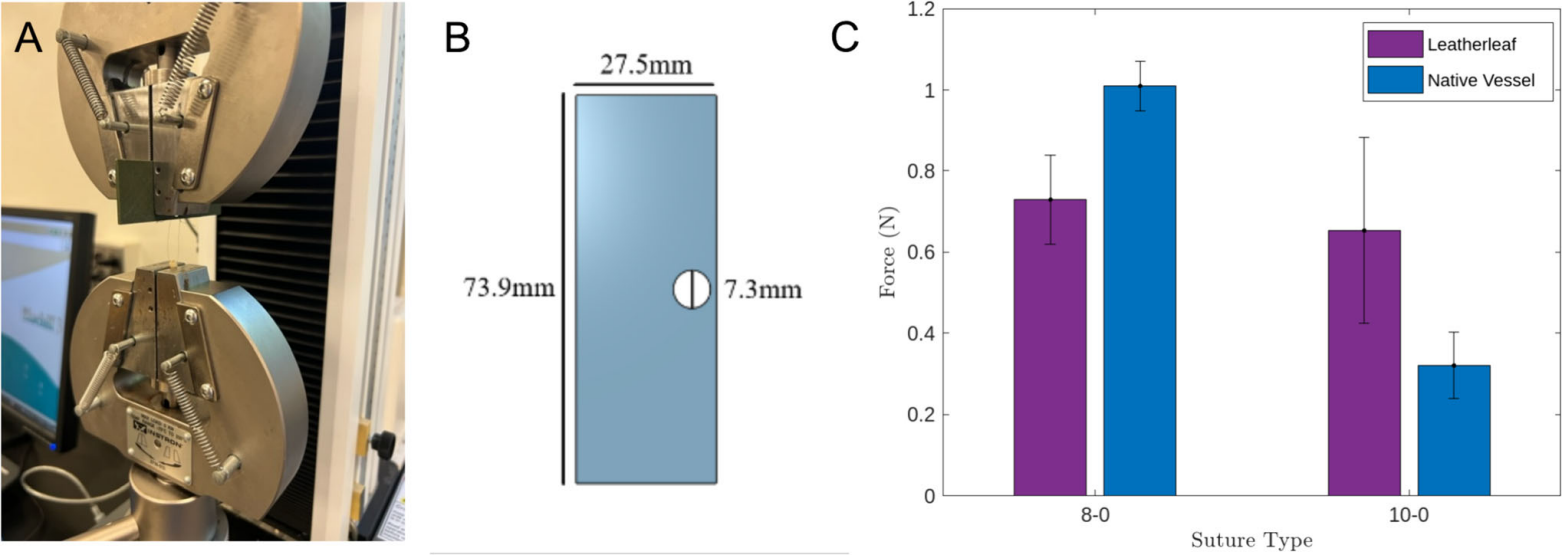
**A** Example of a specimen during a suture retention strength test. **B** CAD model of custom-made 3D stencil used as suture holder. **C** Max load of 8–0 and 10–0 sutures looped through plant-based grafts and native vessels. *p < 0.05 and error bars represent standard deviation (*N* = *3*). One-way ANOVA and post hoc Tukey tests were used to compare data between groups with a *p* value of < 0.05 to determine statistical significance

Suture retention tests were performed on plant-based grafts and rat aorta to compare maximum load of 8–0 and 10–0 Prolene sutures (N = 3). The suture was looped through the center of the graft or aorta, 2 mm below the end, and knotted onto a custom-made suture holder (Fig. 2A, B). The graft or aorta was clamped to one end of the INSTRON, and the suture holder clamped to the other. Force was measured using a 100 N load cell and tension was applied to sutures at a rate of 1 mm/s until a tear in the graft or aorta was observed as previously described [27].

### White cell assay

Fresh whole rat blood was collected and stored in citrate tubes and stored at room temperature for 2 h. Two mL of blood was mixed with 2 mL of PBS, layered on top of 3 mL of Lymphoprep Density Gradient Medium (Sigma-Aldrich), then centrifuged at 400 g for 30 min at 18 ºC. The layer of mononuclear cells was washed in PBS and centrifuged then the cell pellet was transferred to M199 medium. To evaluate the foreign body reaction *in vitro,* 1 million white cells were seeded onto 1.5 × 1.5 cm decellularized leatherleaf viburnum scaffolds that were either acellular or previously seeded with endothelial cells and vascular smooth muscle cells and incubated for 24 h under static conditions (N = 3). Cell density on these scaffolds was evaluated using trypan exclusion and compared to a polystyrene control as previously described [28].

### Statistical analysis

An Anderson–Darling test was used to determine if data were normally distributed. Groups of three or more were compared using one-way ANOVA. If there were significant differences between groups, a post hoc Tukey test was used for pair-wise comparisons of data with a *p* value of < 0.05 to determine statistical significance through Microsoft Excel (Redmond, WA, USA). Unpaired t-tests determined statistical significance (*p* < 0.05) for groups of two through Microsoft Excel. Data are expressed as mean ± standard deviation.

## Results

### Cell analysis

**Fig. 3:**
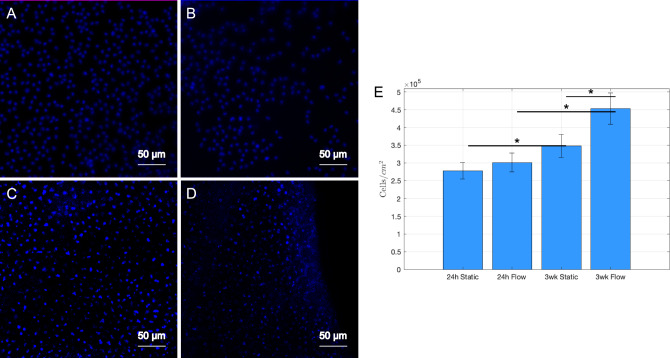
Representative 20 × magnification images of endothelial cells seeded on grafts with **A** 24 h no flow and **B** 24 h flow (*N* = *3*), and **C** 3 week no flow and **D** 3 week flow (*N* = *3*) stained with Hoechst. **E** Cell count for grafts with seeded endothelial cells under static and flow conditions. *p < 0.05 and error bars represent standard deviation. Cells were counted using 3 images per sample (*N* = *9*). One-way ANOVA and post hoc Tukey tests were used to compare data between groups with a *p* value of < 0.05 to determine statistical significance

Grafts coated with fibronectin were seeded with vascular smooth muscle cells and endothelial cells and stained with Hoechst to assess cell performance in the bioreactor system after application of fluid flow (Fig. 3). A post hoc Tukey test showed that endothelial cell density after 24 h of exposure to fluid flow did not change significantly compared to static culture (301,111 ± 23,333 vs. 277,778 ± 26,193 cells/cm^2^). Endothelial cell density significantly increased by 25% after 3 weeks of static culture compared to 24 h of static culture (*p* < 0.05). Endothelial cell density significantly increased by 51% after 3 weeks of treatment with fluid flow (453,703 ± 44,215 cells/cm^2^) compared to 24 h of treatment (301,111 ± 26,193 cells/cm^2^) with fluid flow (*p* < 0.05). Endothelial cell density after 3 weeks of bioreactor treatment with fluid flow increased significantly by 30% compared to 3 weeks of culture under static conditions (*p* < 0.05). The live/dead assay showed 83 ± 6%, 88 ± 5% and 89 ± 2% of vascular smooth muscle cells in the grafts were viable after 24 h or 3 weeks under static culture, or 3 weeks of flow at a density of 21,000 cells/cm^2^, respectively (Online Resource 3). Additionally, the bioreactor maintained infection free culture for the entire 3-week experiment (Online Resource 4).

### Thrombogenicity testing

**Fig. 4:**
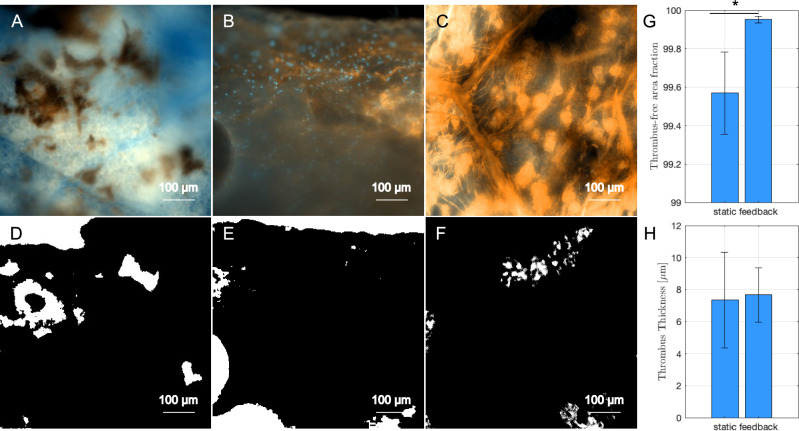
Representative 10 × magnification images of thrombosis testing on **A** acellular grafts, **B** seeded grafts without bioreactor preconditioning and **C** seeded grafts with bioreactor feedback treatment and their corresponding threshold images **D–F**. Quantification of thrombosis through **G** thrombus-free area fraction and **H** thrombus thickness. *p < 0.05 and error bars represent standard deviation. Unpaired *t*-tests were used to determine statistical significance

Thrombus-free area of 24 h bioreactor feedback-treated grafts (Fig. 4A–F) increased significantly from 99.6 ± 0.2% to 99.9 ± 0.02% (*t*-test, *p* < 0.05) compared to seeded grafts without bioreactor treatment, representing a 89% decrease in thrombus area (Fig. 4G). Average thrombus thickness did not change significantly (*p* > 0.05) for grafts with and without bioreactor treatment, with thicknesses of 7.7 ± 1.7 µm versus 7.4 ± 3.0 µm, respectively (Fig. 4H). Moderate thrombosis was observed for acellular grafts, with a thrombus-free area of 90.2 ± 3% and thrombus thickness of 61 ± 11 µm.

### Structural analysis

**Fig. 5:**
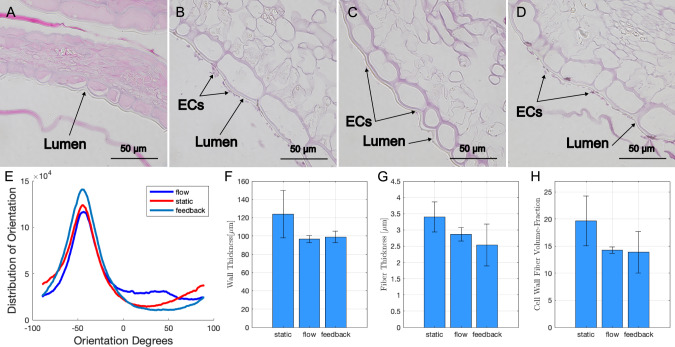
Representative H&E images of grafts at 40 × magnification with **A** no endothelial cells (ECs) and vascular smooth muscle cells, **B** endothelial cells and vascular smooth muscle cells, **C** endothelial cells and vascular smooth muscle cells with flow, **D** endothelial cells and vascular smooth muscle cells with flow and pressure. **E** Distribution of ECM orientation. **F** Wall thickness, **G** fiber thickness and **H** cell wall fiber volume-fraction were measured for 3 images per sample (*N* = *9*). Error bars represent standard deviation

H&E staining confirmed cell removal from SDS-decellularized leatherleaf which was used to construct the 3D grafts (Online Resource 5). These grafts were recellularized with endothelial cells and vascular smooth muscle cells, maintained in static conditions, exposed to fluid flow or fluid flow with a pressure waveform for 24 h. This multilayer ECM structure was preserved for all flow rates and pressures described above (Fig. 5A–D). A one-way ANOVA yielded no significant variations between conditions (*p* > 0.05) for wall thickness, fiber thickness, cell wall fiber volume-fraction and ECM orientation (Fig. 5F–H). Cellulose fiber thickness was 3.4 ± 0.5, 2.9 ± 0.2, and 2.5 ± 0.6 µm for static, flow and feedback conditions, respectively. Cell wall fiber volume-fraction was 14 ± 4% for grafts following bioreactor treatment compared to 20 ± 5% for grafts without treatment, and the fibers aligned preferentially along the circumference of the graft (Fig. 5E).

**Fig. 6:**
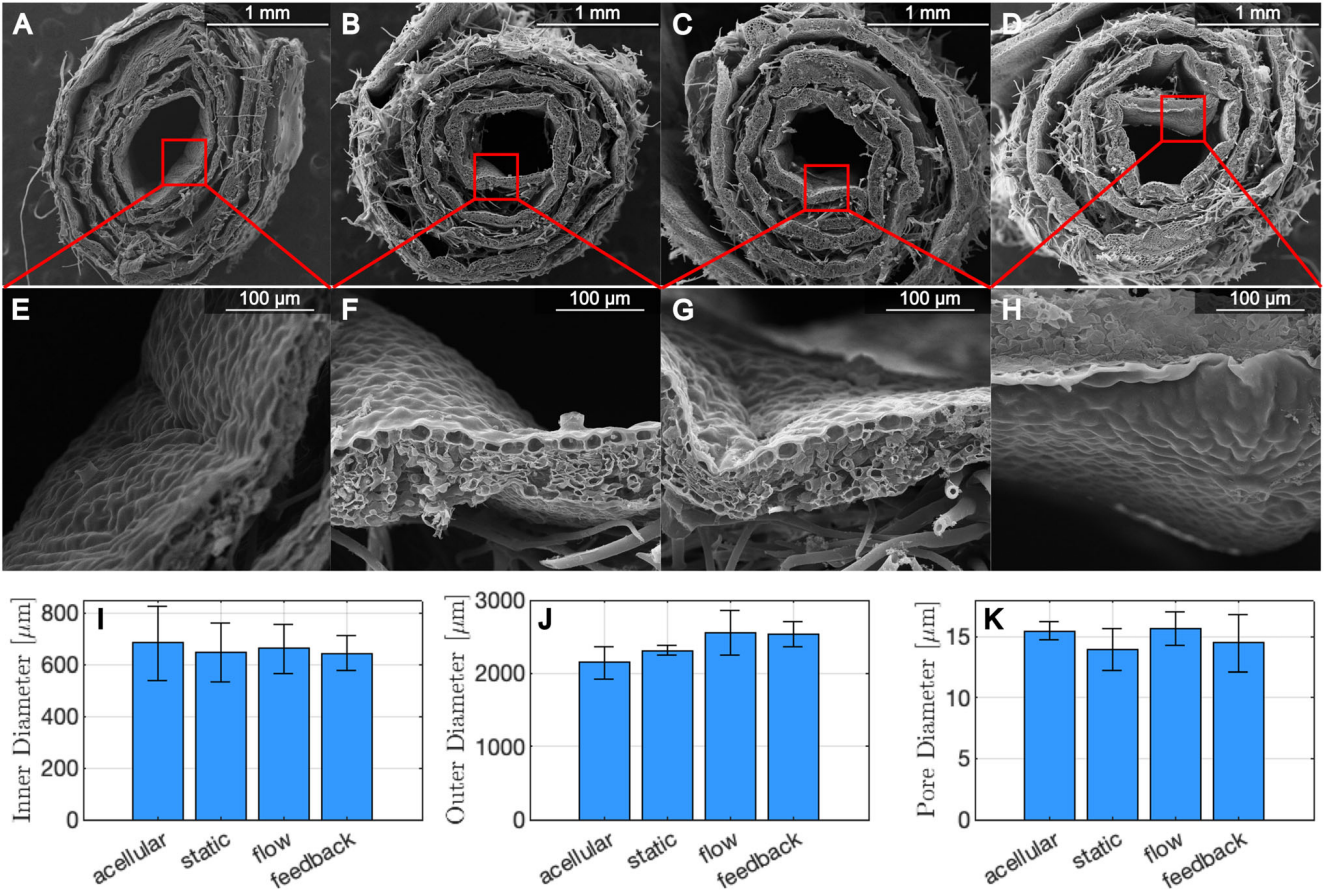
Representative SEM images of **A,E** acellular grafts and seeded grafts under **B,F** static, **C,G** flow and **D,H** flow plus pressure treatment and imaged at magnifications of 110 × and 800 × , respectively. Graft **I** inner diameter, **J** outer diameter and **K** pore diameter were measured for 3 images per sample (*N* = *9*). *p < 0.05 and error bars represent standard deviation

SEM imaging revealed multi-branch trichomes on the abaxial surfaces and a relatively smooth adaxial surface of acellular grafts (Fig. 6A, E). The recellularized grafts (Fig. 6B, F) maintained their multilayer integrity and structure after exposure to fluid flow (Fig. 6C, G) or fluid flow with a pressure waveform (Fig. 6D, H). A post hoc Tukey test showed that outer diameter of the graft increased significantly by 10% (2,535 ± 173 vs. 2,311 ± 64 µm, *p* < 0.05) following bioreactor conditioning with fluid flow and pressure compared to the control (Fig. 6J). Average graft inner diameter ranged from 645 to 683 µm, pore size ranged from 13.9 to 15.4 µm within the spongy mesophyll of each leaf scaffold, and they did not vary significantly between conditions (Fig. 6I, K). The pores on the luminal surface of each graft, which averaged 12 ± 2 µm in diameter, were appropriate for endothelial cell proliferation [22].

### Suture retention

Retention force of 8–0 and 10–0 Prolene sutures was 0.74 ± 0.13 and 0.65 ± 0.28 N for plant-based grafts and 1.01 ± 0.17 and 0.32 ± 0.10 N for rat aorta, respectively (Fig. 2C). A post hoc Tukey test showed that retention force of 10–0 sutures was significantly higher (by 103%, *p* < 0.05) in plant-based grafts compared to the rat aorta control.

### Assessment of immunogenicity

After 24 h in culture, there were 71,000 ± 1,900, 119,800 ± 23,900 and 86,000 ± 3600 white cells/cm^2^ on acellular decellularized leatherleaf, endothelial cell and vascular smooth muscle cell seeded leatherleaf and the polystyrene control, respectively. Notably, a post hoc Tukey test showed that white cell viability increased significantly by 68% (*p* < 0.05) on endothelial cell and vascular smooth muscle cell seeded leatherleaf compared to acellular leatherleaf scaffolds.

## Discussion

Over the last decade, several bioreactors have been developed to expose endothelial cells and vascular smooth muscle cells in vascular grafts to fluid flow and cyclic stretch in order to increase cell performance, reduce blood coagulation and improve vessel patency. This is the first study to investigate the effects of bioreactor conditioning on decellularized plant scaffolds, which have shown recent promise in skeletal, cardiac bone, and vascular tissue engineering. Traditional bioreactor systems often rely on the inherent pulsatility of peristaltic pumps [29] and sometimes incorporate static pressure [30] or longitudinal stretch [31], but this provides limited control over pressure and frequency. These systems often add a silicone tube to the lumen of a vessel to provide support and regulate expansion of the vessel [32]. Song et al. were able to independently subject grafts to different stimuli through a programmed microprocessor unit [21]. However, like most other systems, this was not a fully automated system regulated by feedback control.

Our feedback control is a fully automated, closed-loop system where the solenoid valve responds to pressure changes, and the system’s behavior can be monitored and analyzed through real-time data processing. It is a flexible and adaptable solution for scenarios where precise pressure control is crucial, enabling the study of the effects of higher-pressure waveform frequencies that have not yet been investigated. For example, the feedback system can be programmed to match the heart rate (371.5 bpm or 6.19 Hz) and systolic and diastolic pressure of a rat (84–184 mmHg and 58–145 mmHg, respectively). Unlike traditional bioreactors which might be limited to specific vessel sizes, our system can be tailored to fit any diameter graft from coronary artery vessels to larger peripheral or even aortic vessels. This versatility enables the conditioning of patient-specific grafts, potentially improving the success rate of personalized medicine approaches in vascular surgery.

To better understand the impact of fluid flow on endothelial cells and vascular smooth muscle cell performance in plant-based vascular grafts, the grafts were conditioned for up to 3 weeks in the bioreactor. After 3 weeks of treatment with flow, endothelial cells in the lumen increased significantly in density when compared to 24 h of bioreactor treatment and the 3 week condition without flow. Furthermore, vascular smooth muscle cells in the grafts remained viable after 24 h and viability increased after 3 weeks of bioreactor treatment when compared to static controls. The rate of endothelialization of the plant-based grafts in this study was very fast compared to clincially used expanded polytetrafluoroethylene grafts, which endothelialze at < 10% after 3 weeks [33]. To our knowledge, this is the first time that the effects of physiological shear forces have been evaluated on plant-derived vascular grafts. It is worth noting that the mean shear stress and Reynolds number in these grafts were very similar to that of human coronary arteries [34]. In all conditions, our endothelial cell densities were higher than what was previously reported for human umbilical vein endothelial cells (HUVECs) seeded on luminal surfaces of synthetic vascular grafts [35] and our previous results with seeding of rat endothelial cells on SDS-decellularized leatherleaf and HUVECs on decellularized umbilical arteries without flow [22, 36]. Others have developed bioreactor systems to precondition scaffolds seeded with endothelial cells and observed varying cell counts after mechanical stimulation compared to static culture [37]. Vouyoula et al. showed that a pulsatile pressure of 160/110 mmHg at 1 Hz reduces aortic endothelial cell proliferation [38]. Shin et al. showed reduced HUVEC proliferation when exposed to 140/100 mmHg cylcic pressure for 24 h [39], and found HUVEC proliferation increased in response to 60/20 mmHg at 1 Hz for 24 h. This suggests endothelial cell proliferation in response to cyclic pressure and stretch may be dependent on cell type (arterial versus venous) and magnitude of applied pressures. Several studies have also shown higher surface roughness of vascular graft materials can increase cell attachment, proliferation and differentiation [40]. Salehi et al. previously demonstrated through atomic force microscopy that decellualrized spinach possesed a roughness average of 11.5 nm which promoted stem cell binding and proliferation, and this roughness was consistent with our SEM analysis of decellularized leatherleaf [41].

Due to the prevalence of thrombosis in small-diameter vascular grafts *in vivo*, it is important to assess thrombogenicity of new plant-derived scaffolds in order to determine the impact of endothelialization (described above) and bioreactor pretreatment on graft performance *in vitro*. Our results indicate that endothelialization of plant-derived grafts significantly reduces thrombus formation *in vitro* and the application of fluid flow and pressure can further reduce thrombosis. To the best of our knowledge, no other studies have demonstrated reduced thrombus formation in plant-derived grafts. We observed moderate thrombus thickness in acellular grafts, which was greatly reduced by endothelialization and bioreactor conditioning with 24 h of fluid flow and pressure. Limiting the thrombosis assay to a 30-min incubation time to evaluate the blood-material interaction was necessary in this study to prevent the plateauing of effects caused by the depletion of activation products [42]. Experiments with extended circulation in a Chandler loop model indicated that first contact contributed the biggest effect and that prolonged circulation will not cause further differentiation between materials [43]. Our thrombus-free area measured by fibrin deposition and platelet adhesion was also comparable to that of Fleser et al. Similarly, Fleser et al. showed that treatment of 5 mm polyurethane vascular grafts reduced thrombus free surface in a sheep model from 98 to 47% compared to the control, with a maximum thrombus thickness of 200 µm [44]. Since endothelial cells maintained a high density in our grafts after 3 weeks of application of fluid flow, these cells will be less likely to detach due to high shear flows after implantation which can subsequently help prevent thrombosis *in vivo*. The low thrombosis formation in our grafts suggests that plant-derived materials seeded with endothelial cells are appropriate for overcoming thrombosis in small-diameter vascular grafts, which is a key challenge in vascular graft research. Additionally, this study demonstrates the potential for bioreactor conditioning to increase endothelial cell proliferation and reduce thrombus formation in plant-derived vascular tissues. These strategies could aid in the development of other plant-based grafts, such as decellularized parsley stems [6].

To assess stability and *in vivo* applications of our plant-based grafts following mechanical preconditioning, we quantified maintenance of wall integrity, as shown in our analysis of H&E staining and SEM imaging following decellularization and bioreactor preconditioning. We found that ECM integrity was preserved, as measured by wall thickness, fiber thickness and cell wall fiber volume-fraction. The low cell wall volume-fiber volume fraction of these cellulose scaffolds was similar to that of decellularized apple and has been reported to promote blood vessel formation [45]. The cellulose fiber thickness was also comparable to previous studies, and the pore size was similar to previous reports for parsley and vanilla [46]. Together, these fiber properties could help promote infiltration of cells after implantation. Additionally, the alignment of the cellulose fibers following bioreactor treatment of the grafts is desirable for recreating the non-linear and anisotropic structure of vascular tissue. Fiber orientation in each layer (tunica intima, media and adventia) of native arteries contributes significantly to their mechanical properties. Collagen fibers in the adventitia of human coronary arteries have been shown to orient at angles of ± 67 degrees relative to the circumference, which is very similar to the cellulose fiber orientation we report here [47, 48]. Wall thickness also increased slightly following bioreactor treatment from cell proliferation and matrix deposition, which would help decrease intramural stress in cases of increased blood pressure after implation [49].

Clinical vascular sutures were selected for this study (8–0 and 10–0 polypropylene monofilament sutures) to assess suture retention force as they are used at these sizes for human coronary arerty bypass and rat abdominal aorta interposition, respectively [50, 51]. These sutures are non-absorbable, flexible and have low friction. Suture retention force of our plant-based grafts was within the the vascular transplantation standard of 0.6–1.5 N per needle for both suture sizes [52]. Additionally, the retention force of 10–0 sutures in the plant-based grafts was significantly higher than that of rat aorta. Wall thickness is a critical factor affecting the mechanical strength of tissue-engineered grafts and our grafts demonstrated similar suture retention force as other grafts materials with similar wall thickness [9]. The suture retention forces demonstrated here will help anastomose these grafts during implantation, prevent leakage and withstand forces within the circulatory system. This is the first study to evaulate suture retention force in a plant-derived graft and our findings suggest that plant-based materials will be suitable for use in vascular repair.

Immunogencity of plant-derived scaffolds remains another potential cause for rejection during transplantation. It is still unknown what effects detergents have on plant ECM and how these scaffolds will interact with the innate and adaptive immunity. Modulevsky et al. subcutaneously implanted decellularized apple which exhibited an immune response after 1 week that gradually disappeared within 8 weeks [53]. Our white cell viability improved significantly on decellularized leatherleaf scaffolds seeded with endothelial cells and vascular smooth muscle cells *in vitro*, and was comparable to that of Schutte et al. [28] where viability of monocytes seeded on common biomaterial surfaces was measured by trypan dye exclusion to evaluate immunogoencity of common biomaterials. This increase in white cell viability is important because Roh et al. previously demonstrated that tissue-engineered vascular grafts remodel by recruitment of monocytes via an inflammation-mediated process [54]. Furthermore, others have shown that endothelial cells seeded on fibronectin-coated grafts have shown increased relase of interleukin-6, which promotes vascular smooth muscle cell proliferation and monocyte survival and recruitment [55].

In this paper we present a novel bioreactor design which marks the first time that a bioreactor has been used to precondition decellularized plant scaffolds for tissue repair. Using precise application of fluid flow and pressure at higher frequencies, plant-based grafts cultured in this system showed increased endothelial cell density of up to 51% and reduced thrombus formation within the lumen, and maintenance of viable vascular smooth muscle cells within the inner layers. Extended culture for up to 3 weeks demonstrates the bioreactor’s ability to maintain infection free culture, while improving cell performance. Furthermore, suture retention met the transplantation standard and immunogenicity of the grafts was appropriate to promote vascular remodeling. This study provides insight into how physiological hemodynamic forces can be utilized at specific frequencies to precondition plant-based grafts for vascular repair. Future studies will involve implantation of bioreactor preconditioned grafts into a rat model to determine if mechanical stimuli generated by our feedback system are able to improve long term patency and reduce thrombogenicity of grafts *in vivo*. Additionally, protein content of decellularized plant scaffolds will be analyzed *in vitro* to further understand their potential immunogenicity.

## Supplementary Information

Below is the link to the electronic supplementary material.

Supplementary file 1 (DOCX 3057 kb)

## Data Availability

All data generated or analyzed are included in this article. Further inquiries can be directed to the corresponding author.

## References

[1] Weintraub WS. The economic burden of illness. JAMA Netw Open. 2023;6:e232663–763. 36912842 10.1001/jamanetworkopen.2023.2663

[2] Gershlak JR, Hernandez S, Fontana G, Perreault LR, Hansen KJ, Larson SA, et al. Crossing kingdoms: using decellularized plants as perfusable tissue engineering scaffolds. Biomaterials. 2017;125:13–22. 28222326 10.1016/j.biomaterials.2017.02.011 PMC5388455

[3] Eslami MH, Gangadharan SP, Belkin M, Donaldson MC, Whittemore AD, Conte MS. Monocyte adhesion to human vein grafts: A marker for occult intraoperative injury? J Vasc Surg. 2001;34:923–9. 11700496 10.1067/mva.2001.118590

[4] Moore MJ, Tan RP, Yang N, Rnjak-Kovacina J, Wise SG. Bioengineering artificial blood vessels from natural materials. Trends Biotechnol. 2022;40:693–707. 34887104 10.1016/j.tibtech.2021.11.003

[5] Hadinata IE, Hayward PA, Hare DL, Matalanis GS, Seevanayagam S, Rosalion A, et al. Choice of conduit for the right coronary system: 8-year analysis of radial artery patency and clinical outcomes trial. Ann Thorac Surg. 2009;88:1404–9. 19853082 10.1016/j.athoracsur.2009.06.010

[6] Cevik M, Dikici S. Development of tissue-engineered vascular grafts from decellularized parsley stems. Soft Matter. 2024;20:338–50. 38088147 10.1039/d3sm01236k

[7] Gillette BM, Rossen NS, Das N, Leong D, Wang M, Dugar A, et al. Engineering extracellular matrix structure in 3D multiphase tissues. Biomaterials. 2011;32:8067–76. 21840047 10.1016/j.biomaterials.2011.05.043 PMC3340985

[8] Merna N, Robertson C, La A, George SC. Optical imaging predicts mechanical properties during decellularization of cardiac tissue. Tissue Eng Part C Methods. 2013;19:802–9. 23469868 10.1089/ten.tec.2012.0720 PMC3751370

[9] Meng X, Wang X, Jiang Y, Zhang B, Li K, Li Q. Suture retention strength of P (LLA-CL) tissue-engineered vascular grafts. RSC Adv. 2019;9:21258–64. 35521332 10.1039/c9ra04529e PMC9065988

[10] Yang S, Leong KF, Du Z, Chua CK. The design of scaffolds for use in tissue engineering. Part I Tradit factor Tissue Eng. 2001;7:679–89. 10.1089/107632701753337645 11749726

[11] Lock A, Cornish J, Musson DS. The role of in vitro immune response assessment for biomaterials. J Funct Biomater. 2019;10:31. 31336893 10.3390/jfb10030031 PMC6787714

[12] Bilodeau K, Couet F, Boccafoschi F, Mantovani D. Design of a perfusion bioreactor specific to the regeneration of vascular tissues under mechanical stresses. Artif Organs. 2005;29:906–12. 16266305 10.1111/j.1525-1594.2005.00154.x

[13] Bacci C, Wong V, Barahona V, Merna N. Cardiac and lung endothelial cells in response to fluid shear stress on physiological matrix stiffness and composition. Microcirculation. 2021;28:e12659. 32945052 10.1111/micc.12659

[14] Pan S. Molecular mechanisms responsible for the atheroprotective effects of laminar shear stress. Antioxid Redox Signal. 2009;11:1669–82. 19309258 10.1089/ars.2009.2487 PMC2842586

[15] Eoh JH, Shen N, Burke JA, Hinderer S, Xia Z, Schenke-Layland K, et al. Enhanced elastin synthesis and maturation in human vascular smooth muscle tissue derived from induced-pluripotent stem cells. Acta Biomater. 2017;52:49–59. 28163239 10.1016/j.actbio.2017.01.083

[16] Kural MH, Dai G, Niklason LE, Gui L. An ex vivo vessel injury model to study remodeling. Cell Transplant. 2018;27:1375–89. 30095004 10.1177/0963689718792201 PMC6168986

[17] Wolf F, Rojas González DM, Steinseifer U, Obdenbusch M, Herfs W, Brecher C, et al. VascuTrainer: a mobile and disposable bioreactor system for the conditioning of tissue-engineered vascular grafts. Ann Biomed Eng. 2018;46:616–26. 29340931 10.1007/s10439-018-1977-y

[18] van Haaften EE, Wissing TB, Rutten MC, Bulsink JA, Gashi K, van Kelle MA, et al. Decoupling the effect of shear stress and stretch on tissue growth and remodeling in a vascular graft. Tissue Eng Part C Methods. 2018;24:418–29. 29877143 10.1089/ten.TEC.2018.0104

[19] Zaucha MT, Raykin J, Wan W, Gauvin R, Auger FA, Germain L, et al. A novel cylindrical biaxial computer-controlled bioreactor and biomechanical testing device for vascular tissue engineering. Tissue Eng Part A. 2009;15:3331–40. 19385725 10.1089/ten.tea.2008.0369 PMC2792052

[20] Lee SJ, Liu J, Oh SH, Soker S, Atala A, Yoo JJ. Development of a composite vascular scaffolding system that withstands physiological vascular conditions. Biomaterials. 2008;29:2891–8. 18400292 10.1016/j.biomaterials.2008.03.032

[21] Song L, Zhou Q, Duan P, Guo P, Li D, Yuan X, Li S, Luo F, Zhang Z. Successful development of small diameter tissue-engineering vascular vessels by our novel integrally designed pulsatile perfusion-based bioreactor. PLoS One. 2012;7:e42569. 22880036 10.1371/journal.pone.0042569 PMC3411804

[22] Gorbenko N, Rinaldi G, Sanchez A, Merna N. Small-caliber vascular grafts engineered from decellularized leaves and cross-linked gelatin. Tissue Eng Part A. 2023;29:397–409. 37053092 10.1089/ten.tea.2022.0223 PMC10354733

[23] Fontana G, Gershlak J, Adamski M, Lee JS, Matsumoto S, Le HD, et al. Biofunctionalized plants as diverse biomaterials for human cell culture. Adv Healthc Mater. 2017;6:1601225. 10.1002/adhm.201601225 PMC5490445 28319334

[24] Luo Y, Owens D, Mulder G, McVey A, Fisher T. Blood pressure characterization of hypertensive and control rats for cardiovascular studies. AHA, Atlanta: Charles River. 2008.

[25] Blanquer A, Careta O, Anido-Varela L, Aranda A, Ibáñez E, Esteve J, et al. Biocompatibility and electrical stimulation of skeletal and smooth muscle cells cultured on piezoelectric nanogenerators. Int J Mol Sci. 2021;23:432. 35008860 10.3390/ijms23010432 PMC8745485

[26] Lehmann M, Schoeman RM, Krohl PJ, Wallbank AM, Samaniuk JR, Jandrot-Perrus M, et al. Platelets drive thrombus propagation in a hematocrit and glycoprotein VI–dependent manner in an in vitro venous thrombosis model. Arterioscler Thromb Vasc Biol. 2018;38:1052–62. 29472230 10.1161/ATVBAHA.118.310731 PMC5920765

[27] Pensalfini M, Meneghello S, Lintas V, Bircher K, Ehret AE, Mazza E. The suture retention test, revisited and revised. J Mech Behav Biomed Mater. 2018;77:711–7. 28867371 10.1016/j.jmbbm.2017.08.021

[28] Schutte RJ, Parisi-Amon A, Reichert WM. Cytokine profiling using monocytes/macrophages cultured on common biomaterials with a range of surface chemistries. J Biomed Mater Res A. 2009;88:128–39. 18260130 10.1002/jbm.a.31863 PMC4070304

[29] Bono N, Meghezi S, Soncini M, Piola M, Mantovani D, Fiore GB. A dual-mode bioreactor system for tissue engineered vascular models. Ann Biomed Eng. 2017;45:1496–510. 28224370 10.1007/s10439-017-1813-9

[30] Tondreau MY, Laterreur V, Gauvin R, Vallières K, Bourget J-M, Lacroix D, et al. Mechanical properties of endothelialized fibroblast-derived vascular scaffolds stimulated in a bioreactor. Acta Biomater. 2015;18:176–85. 25749291 10.1016/j.actbio.2015.02.026

[31] Mironov V, Kasyanov V, McAllister K, Oliver S, Sistino J, Markwald R. Perfusion bioreactor for vascular tissue engineering with capacities for longitudinal stretch. J Craniofac Surg. 2003;14:340–7. 12826805 10.1097/00001665-200305000-00012

[32] Seliktar D, Nerem RM, Galis ZS. Mechanical strain-stimulated remodeling of tissue-engineered blood vessel constructs. Tissue Eng. 2003;9:657–66. 13678444 10.1089/107632703768247359

[33] Pektok E, Nottelet B, Tille J-C, Gurny R, Kalangos A, Moeller M, et al. Degradation and healing characteristics of small-diameter poly (ε-caprolactone) vascular grafts in the rat systemic arterial circulation. Circulation. 2008;118:2563–70. 19029464 10.1161/CIRCULATIONAHA.108.795732

[34] Pandey R, Yadav PK. Effect of reynolds number and blood viscosity models on the left coronary artery with multiple stenoses. Phys Fluids. 2022;34:091903.

[35] Dong X, Yuan X, Wang L, Liu J, Midgley AC, Wang Z, et al. Construction of a bilayered vascular graft with smooth internal surface for improved hemocompatibility and endothelial cell monolayer formation. Biomaterials. 2018;181:1–14. 30056334 10.1016/j.biomaterials.2018.07.027

[36] Wong V, Gada S, Singh M, Merna N. The development of small-caliber vascular grafts using human umbilical artery: an evaluation of methods. Tissue Eng Part C: Methods. 2023;29:1–10. 36322709 10.1089/ten.TEC.2022.0144

[37] Melchiorri AJ, Bracaglia LG, Kimerer LK, Hibino N, Fisher JP. In vitro endothelialization of biodegradable vascular grafts via endothelial progenitor cell seeding and maturation in a tubular perfusion system bioreactor. Tissue Eng Part C Methods. 2016;22:663–70. 27206552 10.1089/ten.tec.2015.0562 PMC4943466

[38] Vouyouka AG, Powell RJ, Ricotta J, Chen H, Dudrick DJ, Sawmiller CJ, et al. Ambient pulsatile pressure modulates endothelial cell proliferation. J Mol Cell Cardiol. 1998;30:609–15. 9515036 10.1006/jmcc.1997.0625

[39] Shin HY, Gerritsen ME, Bizios R. Regulation of endothelial cell proliferation and apoptosis by cyclic pressure. Ann Biomed Eng. 2002;30:297–304. 12051615 10.1114/1.1458595

[40] Milleret V, Hefti T, Hall H, Vogel V, Eberli D. Influence of the fiber diameter and surface roughness of electrospun vascular grafts on blood activation. Acta Biomater. 2012;8:4349–56. 22842036 10.1016/j.actbio.2012.07.032

[41] Salehi A, Mobarhan MA, Mohammadi J, Shahsavarani H, Shokrgozar MA, Alipour A. Efficient mineralization and osteogenic gene overexpression of mesenchymal stem cells on decellularized spinach leaf scaffold. Gene. 2020;757:144852. 32599019 10.1016/j.gene.2020.144852

[42] Mason R, Zucker W, Shinoda B, Chuang H, Kingdon H, Clark H. Study of the reactions of blood with artificial surfaces use of the thrombogenerator. Lab Invest J Tech Methods Path. 1974;31:143–55. 4369244

[43] Stevens KN, Aldenhoff YB, van der Veen FH, Maessen JG, Koole LH. Bioengineering of improved biomaterials coatings for extracorporeal circulation requires extended observation of blood-biomaterial interaction under flow. Biomed Res Int. 2007;2007:029464. 10.1155/2007/29464 PMC2246072 18317517

[44] Fleser PS, Nuthakki VK, Malinzak LE, Callahan RE, Seymour ML, Reynolds MM, et al. Nitric oxide–releasing biopolymers inhibit thrombus formation in a sheep model of arteriovenous bridge grafts. J Vasc Surg. 2004;40:803–11. 15472611 10.1016/j.jvs.2004.07.007

[45] Feng B, Jinkang Z, Zhen W, Jianxi L, Jiang C, Jian L, et al. The effect of pore size on tissue ingrowth and neovascularization in porous bioceramics of controlled architecture in vivo. Biomed Mater. 2011;6:015007. 21206002 10.1088/1748-6041/6/1/015007

[46] Zhu Y, Zhang Q, Wang S, Zhang J, Fan S, Lin X. Current advances in the development of decellularized plant extracellular matrix. Front Bioeng Biotechnol. 2021;9:650. 10.3389/fbioe.2021.712262 PMC8335482 34368105

[47] Holzapfel GA, Sommer G, Gasser CT, Regitnig P. Determination of layer-specific mechanical properties of human coronary arteries with nonatherosclerotic intimal thickening and related constitutive modeling. Am J Physiol Heart Circ Physiol. 2005;289:H2048–58. 16006541 10.1152/ajpheart.00934.2004

[48] Chen H, Liu Y, Slipchenko MN, Zhao X, Cheng J-X, Kassab GS. The layered structure of coronary adventitia under mechanical load. Biophys J. 2011;101:2555–62. 22261042 10.1016/j.bpj.2011.10.043 PMC3297804

[49] Humphrey JD. Mechanisms of arterial remodeling in hypertension: coupled roles of wall shear and intramural stress. Hypertension. 2008;52:195–200. 18541735 10.1161/HYPERTENSIONAHA.107.103440 PMC2753501

[50] Lee MK, Song JY, Kim TY, Kim JH, Choi JB, Kuh JH. Simple anastomotic techniques for coronary artery bypass surgery in patients with small coronary arteries or a marked size discrepancy between the coronary artery and graft. Korean j thorac cardiovasc surg. 2016;49:485. 27965931 10.5090/kjtcs.2016.49.6.485 PMC5147479

[51] de Valence S, Tille J-C, Mugnai D, Mrowczynski W, Gurny R, Möller M, et al. Long term performance of polycaprolactone vascular grafts in a rat abdominal aorta replacement model. Biomaterials. 2012;33:38–47. 21940044 10.1016/j.biomaterials.2011.09.024

[52] Billiar K, Murray J, Laude D, Abraham G, Bachrach N. Effects of carbodiimide crosslinking conditions on the physical properties of laminated intestinal submucosa. J Biomed Mater Res B Appl Biomater. 2001;56:101–8. 10.1002/1097-4636(200107)56:1<101::aid-jbm1074>3.0.co;2-6 11309796

[53] Modulevsky DJ, Cuerrier CM, Pelling AE. Biocompatibility of subcutaneously implanted plant-derived cellulose biomaterials. PLoS ONE. 2016;11:e0157894. 27328066 10.1371/journal.pone.0157894 PMC4915699

[54] Roh JD, Sawh-Martinez R, Brennan MP, Jay SM, Devine L, Rao DA, et al. Tissue-engineered vascular grafts transform into mature blood vessels via an inflammation-mediated process of vascular remodeling. Proc Natl Acad Sci U S A. 2010;107:4669–74. 20207947 10.1073/pnas.0911465107 PMC2842056

[55] Roca H, Varsos ZS, Sud S, Craig MJ, Ying C, Pienta KJ. CCL2 and interleukin-6 promote survival of human CD11b+ peripheral blood mononuclear cells and induce M2-type macrophage polarization. J Biol Chem. 2009;284:34342–54. 19833726 10.1074/jbc.M109.042671 PMC2797202

