# Supplementary material for: Perfusion Bioreactor Conditioning of Small-diameter Plant-based Vascular Grafts
Source: Tissue Eng Regen Med. 2024 Oct 1;21(8):1189–201. doi: 10.1007/s13770-024-00670-0 (PMC11589060; doi:10.1007/s13770-024-00670-0)
Supplement: Supplementary file 1 — (DOCX 3057 kb) [file 13770_2024_670_MOESM1_ESM.docx]

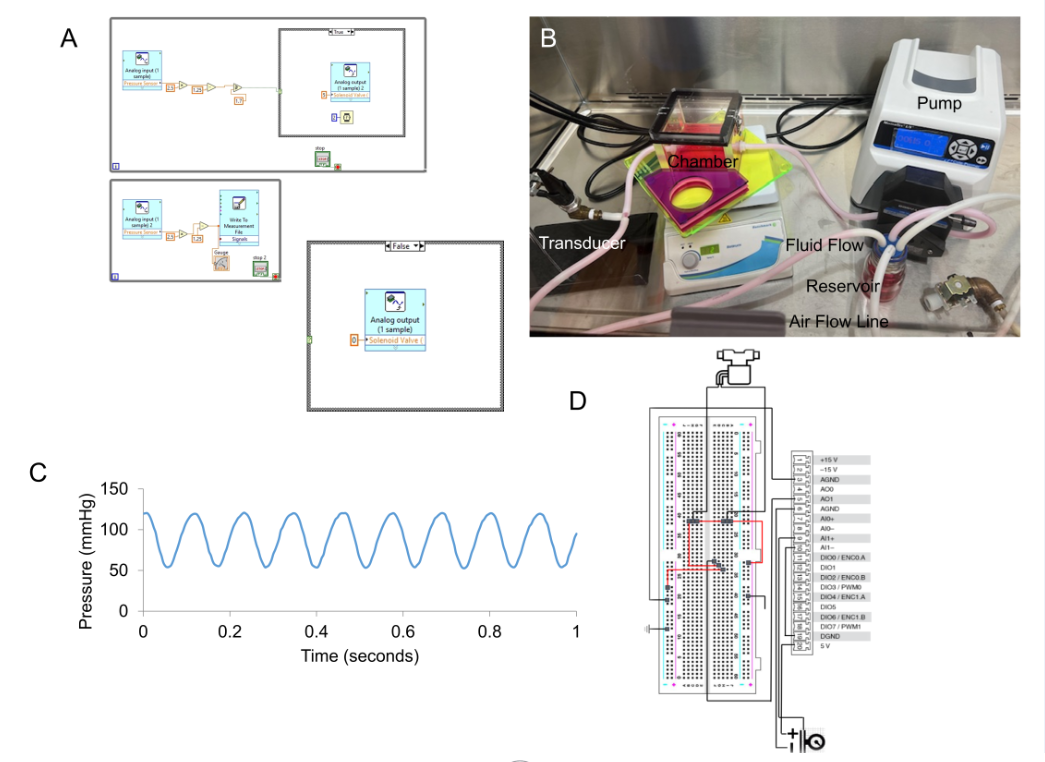
**Online Resource 1. A**LabVIEW block diagram of bioreactor feedback system. **B** Representative images of bioreactor system setup in a biosafety cabinet. **C** Representation of pressure waveform for bioreactor feedback system. **D** Representative image of myRIO connections where red indicates connections within breadboard.


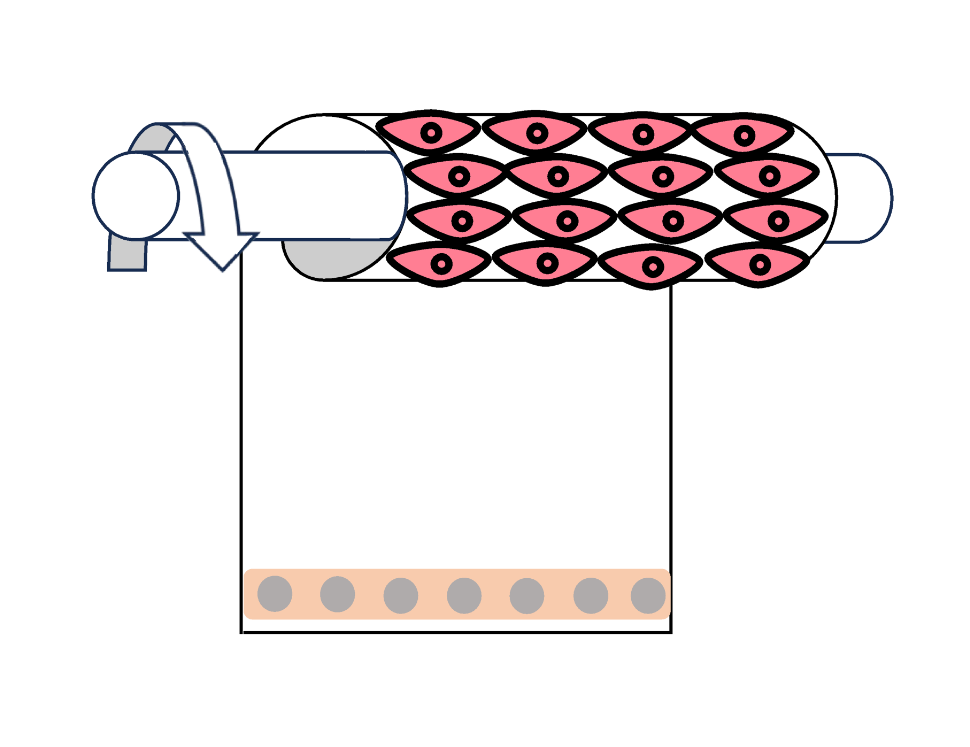
**Online Resource 2.** Illustration of fabrication process for 3D graft. Schematic of SDS-leatherleaf being seeded with vascular smooth muscle cells and rolled counter-clockwise onto a 1mm rod. The orange represents the addition of gelatin and the gray circles are glutaraldehyde.


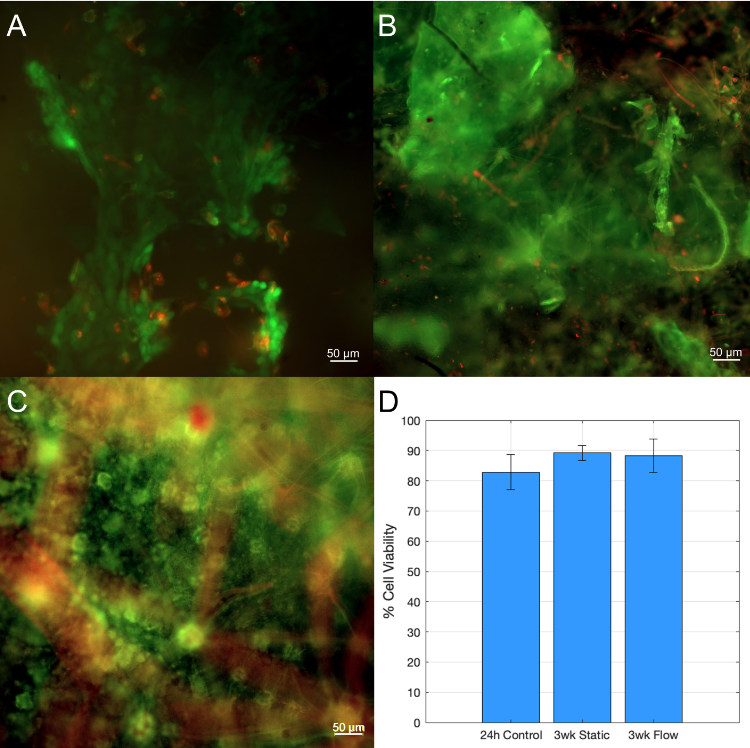
**Online Resource 3.** Live/dead assay for **A** vascular smooth muscle cells on coverslips (control), and on vascular grafts after **B** 24 h, **C** 3 weeks under flow in bioreactor, and **D** percent cell viability (*N=9*).


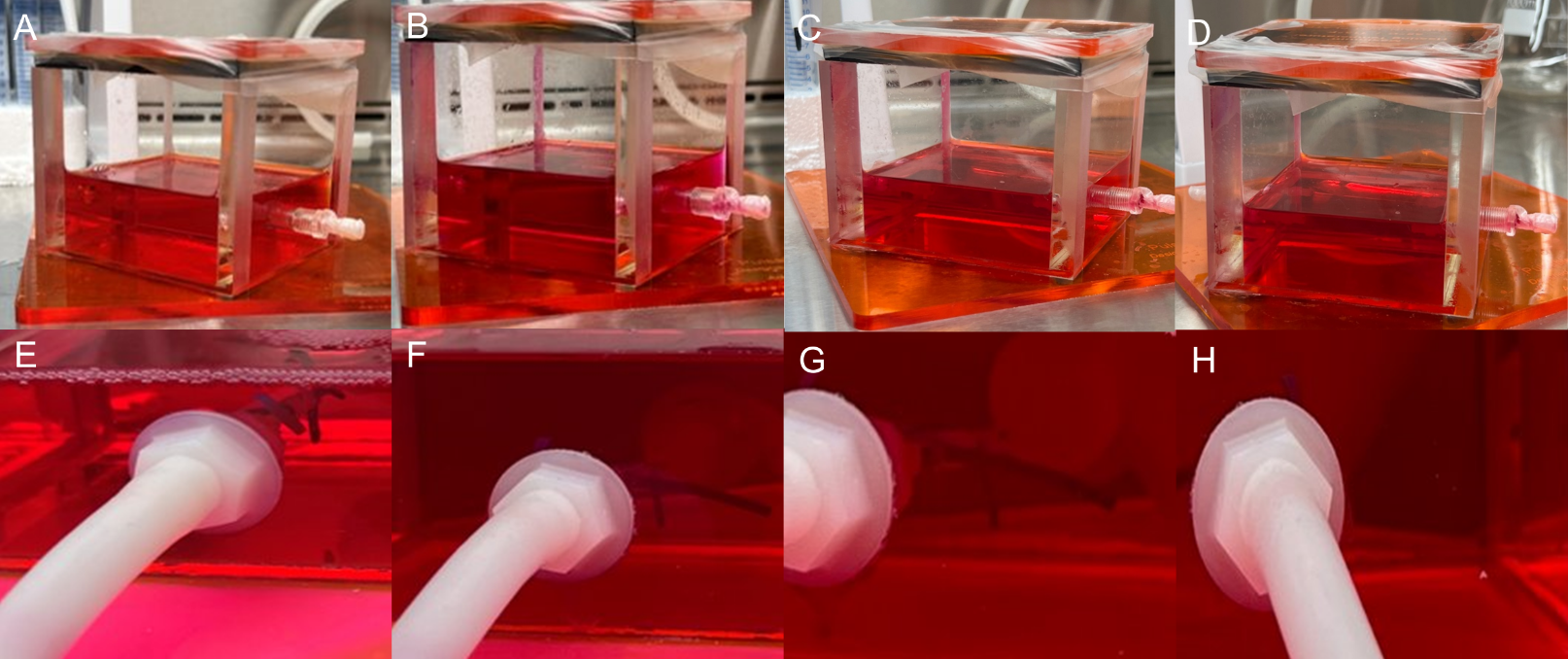
**Online Resource 4.** Representative images of media in the bioreactor chamber with no flow and flow at **A,E** 0 weeks, **B,F** 1 week, **C,G** 2 weeks and **D,H** 3 weeks, respectively.


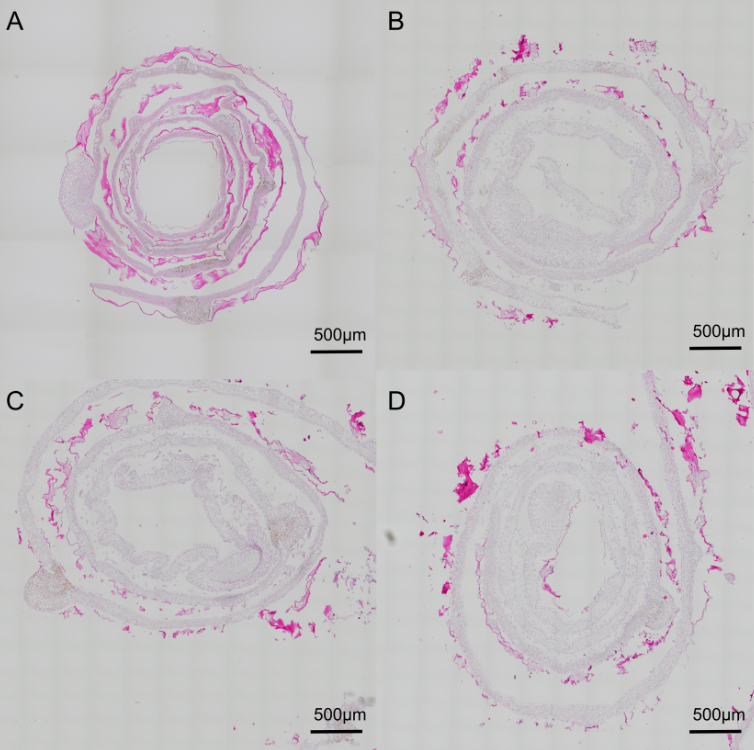
**Online Resource 5.** Representative H&E tile images of grafts at 40× magnification with **A** no endothelial cells and vascular smooth muscle cells, **B** endothelial cells and vascular smooth muscle cells, **C** endothelial cells and vascular smooth muscle cells with 24 h flow, **D** endothelial cells and vascular smooth muscle cells with 24 h flow and pressure.
